# Impact of patient centering in CT on organ dose and the effect of using a positioning compensation system: Evidence from OSLD measurements in postmortem subjects

**DOI:** 10.1002/acm2.12594

**Published:** 2019-05-02

**Authors:** Izabella Barreto, Rebecca Lamoureux, Catherine Olguin, Nathan Quails, Nathalie Correa, Lynn Rill, Manuel Arreola

**Affiliations:** ^1^ Department of Radiology University of Florida College of Medicine Gainesville FL USA; ^2^ Department of Radiology University of New Mexico Albuquerque NM USA

**Keywords:** CT dosimetry, CT imaging, mis‐centering, organ doses, patient centering

## Abstract

The purpose of this study was to investigate the frequency and impact of vertical mis‐centering on organ doses in computed tomography (CT) exams and evaluate the effect of a commercially available positioning compensation system (PCS). Mis‐centering frequency and magnitude was retrospectively measured in 300 patients examined with chest‐abdomen‐pelvis CT. Organ doses were measured in three postmortem subjects scanned on a CT scanner at nine different vertical table positions (maximum shift ± 4 cm). Organ doses were measured with optically stimulated luminescent dosimeters inserted within organs. Regression analysis was performed to determine the correlation between organ doses and mis‐centering. Methods were repeated using a PCS that automatically detects the table offset to adjust tube current output accordingly. Clinical mis‐centering was >1 cm in 53% and 21% of patients in the vertical and lateral directions, respectively. The 1‐cm table shifts resulted in organ dose differences up to 8%, while 4‐cm shifts resulted in organ dose differences up to 35%. Organ doses increased linearly with superior table shifts for the lung, colon, uterus, ovaries, and skin (*R* ^2^ = 0.73–0.99, *P* < 0.005). When the PCS was utilized, organ doses decreased with superior table shifts and dose differences were lower (average 5%, maximum 18%) than scans performed without PCS (average 9%, maximum 35%) at all table shifts. Mis‐centering occurs frequently in the clinic and has a significant effect on patient dose. While accurate patient positioning remains important for maintaining optimal imaging conditions, a PCS has been shown to reduce the effects of patient mis‐centering.

## 1 INTRODUCTION

Modern computed tomography (CT) scanners are equipped with several technological innovations that serve to optimize radiation dose.^1^, ^2^ Among these technologies, automatic tube current modulation (TCM) adjusts the tube current to the specific size and shape of the patient in order to produce diagnostic image quality with minimal radiation exposure to the patient.^3^, ^4^, ^5^ CT scanners also utilize bowtie filters that shape the x‐ray beam to compensate for variations in patient attenuation.^6^, ^7^ In the thickest, central, areas of the patient, a thinner segment of the bowtie filter is used to allow for maximum beam intensity through the anatomical regions with higher attenuation, and in the peripheral areas of the patient, a thicker segment of the filter is used to reduce the beam intensity through the anatomical regions with lower attenuation.

The optimal function of a bowtie filter and TCM techniques require that the patient is centered appropriately in the CT gantry.^8^, ^9^, ^10^, ^11^, ^12^, ^13^, ^14^ When a patient is placed on the CT table, the technologist should attempt to position the patient in the center of the gantry using the gantry‐mounted laser system. This includes aligning the midline of the patient (from the nose to the pubic symphysis) with the central laser and changing the table height so that the center of mass of the anatomy to be scanned is in the center of the gantry. Next, the technologist acquires a localizer radiograph, which serves to measure patient attenuation for proper TCM, as well as help verify correct patient positioning. If necessary, the technologist should correct the patient's position and acquire a new localizer radiograph.

However, studies have shown that technologists do not always correctly center the patient within the gantry.^9^, ^10^, ^11^ This is because patients are not perfectly cylindrical, and it can be difficult to define the center of the patient, especially those who are not lying flat due to physical constraints or examination requirements. Inaccurate centering of the patient in the gantry affects the attenuation of the x‐ray beam and apparent size of the patient, affecting radiation dose and image noise.^11^, ^12^, ^13^, ^14^

**Figure 1:**
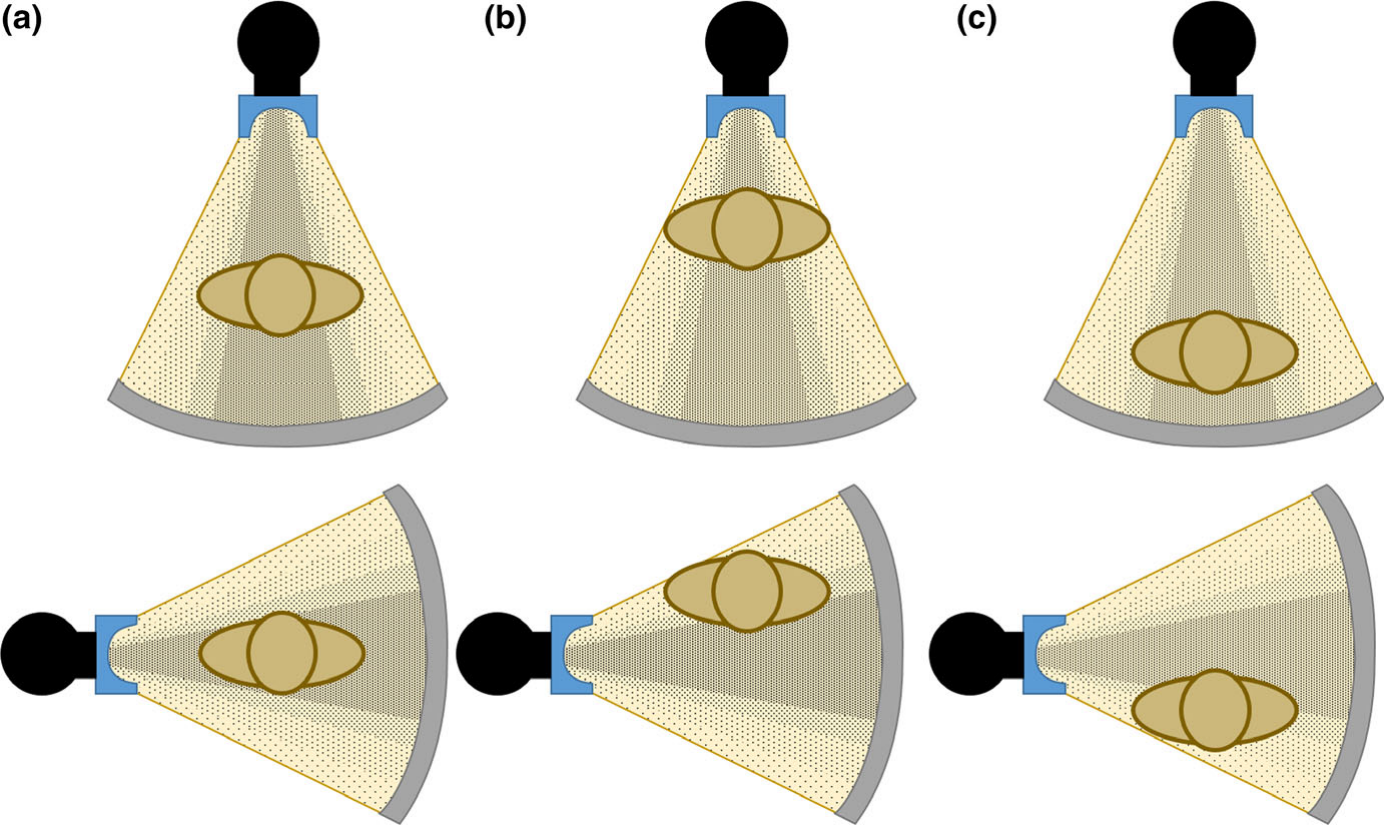
X‐ray beam intensity output from a bow‐tie filter when the x‐ray tube is in the anterior (top) and lateral (bottom) positions with the patient (a) centered in the gantry, (b) shifted anteriorly from center, and (c) shifted posteriorly from center.

The bow‐tie filter shapes the intensity of the x‐ray beam assuming that the thickest region of the patient is located in the center of the beam. If the patient is correctly placed in the center of the gantry, the center of the patient will receive the maximum x‐ray intensity when the x‐ray tube is rotating in the gantry while scanning. This is shown in Fig. 1(a) with the x‐ray tube in the anterior and lateral position. However, if the patient is shifted away from center, the patient will receive a different dose distribution. If the patient is shifted anteriorly away from center, as shown in Fig. 1(b), the anterior organs will receive higher dose when the x‐ray tube is in the anterior position, but they will also receive lower dose when the x‐ray tube is in the posterior position, compared to when the patient was centered. Alternatively, if the patient is shifted posteriorly away from center, as shown in Fig. 1(c), the anterior organs will receive lower dose when the x‐ray tube is in the anterior position, and higher dose when the x‐ray tube is in the posterior position, compared to when the patient was centered. Furthermore, in either the anterior or posterior mis‐centering scenarios, the patient is shifted away from the center and towards the thicker regions of the bow‐tie filter when the x‐ray tube is in the lateral positions. This results in a reduced x‐ray intensity to the majority of the patient's anatomy, as shown in the bottom diagrams of Figs. 1(b) and 1(c). As a result, patient centering is necessary for optimal dose management.^8^, ^9^, ^10^, ^11^, ^12^, ^13^, ^14^

**Figure 2:**
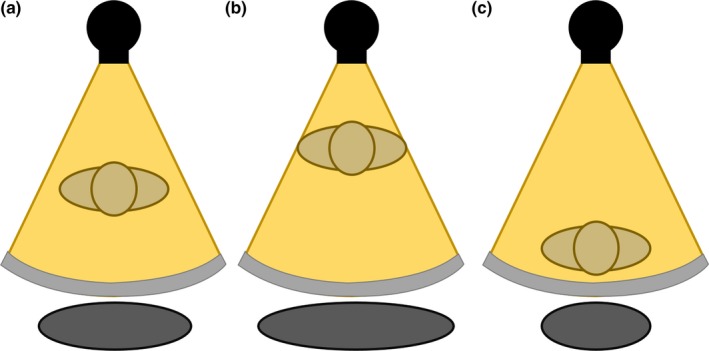
Patient size represented by the localizer radiograph due to magnification when the patient is (a) centered in the gantry, (b) shifted anteriorly from center, and (c) shifted posteriorly from center. The apparent patient size is incorrectly represented as too large in b and too small in c.

Furthermore, if a patient is positioned at the center of the gantry, the magnification in the localizer radiograph is accurate, as shown in Fig. 2(a), and therefore the TCM system will function optimally. However, if the patient is positioned too close to the x‐ray source, the localizer radiograph will experience greater magnification, leading to an overestimation of patient size, as shown in Fig. 2(b). This causes the TCM system to transmit a higher tube current, resulting in an unnecessary increase in radiation dose. Alternatively, if the patient is positioned too far from the x‐ray source, the localizer radiograph will be under‐magnified, shown in Fig. 2(c), resulting in underestimation of attenuation and reduced tube current, potentially resulting in noisy images.

**Figure 3:**
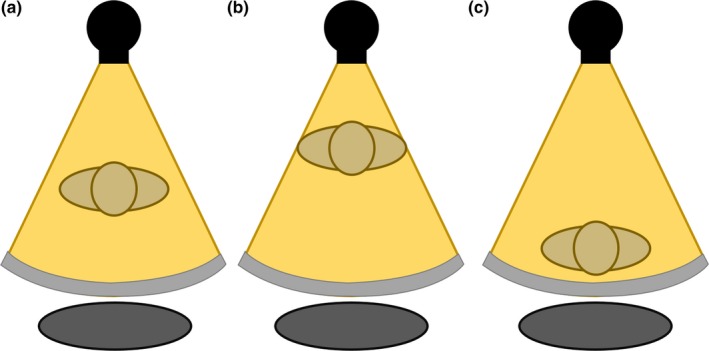
Patient size is corrected by the positioning compensation system when the patient is (a) centered in the gantry, (b) shifted anteriorly from center, and (c) shifted posteriorly from center.

In order to compensate for such magnification effects, a commercially available positioning compensation system (PCS; Auto Couch Height Positioning Compensation, Canon Medical Systems, Otawara, Japan) has been integrated to work with TCM (Sure Exposure 3D, Canon Medical Systems, Otawara, Japan).^15^ This system is a vendor‐proprietary software included in the scanner that automatically detects the offset between the patient's position and the center of the gantry in order to estimate accurate patient size and attenuation.^15^ Figure 3 shows that PCS corrects the magnification effects in the localizer radiograph when the patient is positioned too close [Fig. 3(b)] or too far [Fig. 3(c)] from the x‐ray tube, matching the ideal magnification when the patient is centered [Fig. 3(a)]. The PCS does not alter the appearance of the patient size in the localizer radiograph, it instead communicates with the TCM system that the patient is actually smaller (or larger) than that represented in the localizer radiograph, and in turn, the TCM system produces new tube current maps in the x, y, and z planes using the corrected patient size provided by the PCS. As a result, using the corrected patient attenuation information, the PCS software can remove the effects of magnification and communicate with the TCM system to deliver proper tube current values, even for mis‐centered patients.

The purpose of this study was to investigate the frequency and impact of mis‐centering on organ dose with three specific aims: (a) Investigate the frequency and magnitude of patient mis‐centering in our hospital; (b) Demonstrate the impact of vertical mis‐centering on organ dose in CT using optically stimulated luminescent dosimeters (OSLDs) inserted within postmortem subjects; and (c) Determine whether a PCS can mitigate these effects.

## 2 MATERIALS AND METHODS

### 2.1 Clinical frequency and magnitude of positioning errors

This retrospective study was approved by our institutional review board with waiver of informed consent and was in compliance with the Health Insurance Portability and Accountability Act (HIPAA). This study included 300 patients (age range, 18–94 yr; mean age, 46 yr) who received a Chest‐Abdomen‐Pelvis (CAP) CT examination at our hospital between May 2014 and May 2015. Patients whose abdomens were not fully included within the scan field of view were excluded from the study. All patients were clinically positioned in the CT scanner gantry by CT technologists using the scanner‐mounted laser guidance system.

Studies were viewed on our picture archiving and communication system (PACS) viewing software (Visage Imaging, Inc., Richmond, Vic., Australia). The central axial image of the image series was used to identify each patient's anatomical center in the gantry. To find the center of the cross‐sectional anatomy of each patient, the anteroposterior (AP) diameter of each patient's outermost anatomy was measured and then the lateral (LAT) diameter was measured at half the AP diameter. The half‐length of these two diameters identified the center of the patient. This center was compared to the center of the gantry shown within the image viewing software, and the discrepancy between the two was measured to find the mis‐centering distance in both the vertical and lateral directions. Patients were considered to be perfectly centered in the gantry, and labeled as having zero shift, if their shift from the center of the gantry was <0.5 mm. All mis‐centering distances >0.5 mm were recorded and later binned into 1‐cm groups. Results were analyzed to determine the frequency and magnitude of clinical mis‐centering. Frequency and magnitude were also compared between females and males to investigate whether breasts in females reduced positioning errors. The magnitude of clinical mis‐centering was also correlated with patient effective diameters to determine whether larger patients were mis‐centered by greater magnitudes due to the potential increased difficulty in visually determining the center of a larger patient compared to a smaller patient. The effective diameter was measured for each patient as the square root of the product of their measured AP and LAT diameters, as described in Report 204 of the American Association of Physicists in Medicine.^16^

### 2.2 Organ dosimetry

**Figure 4:**
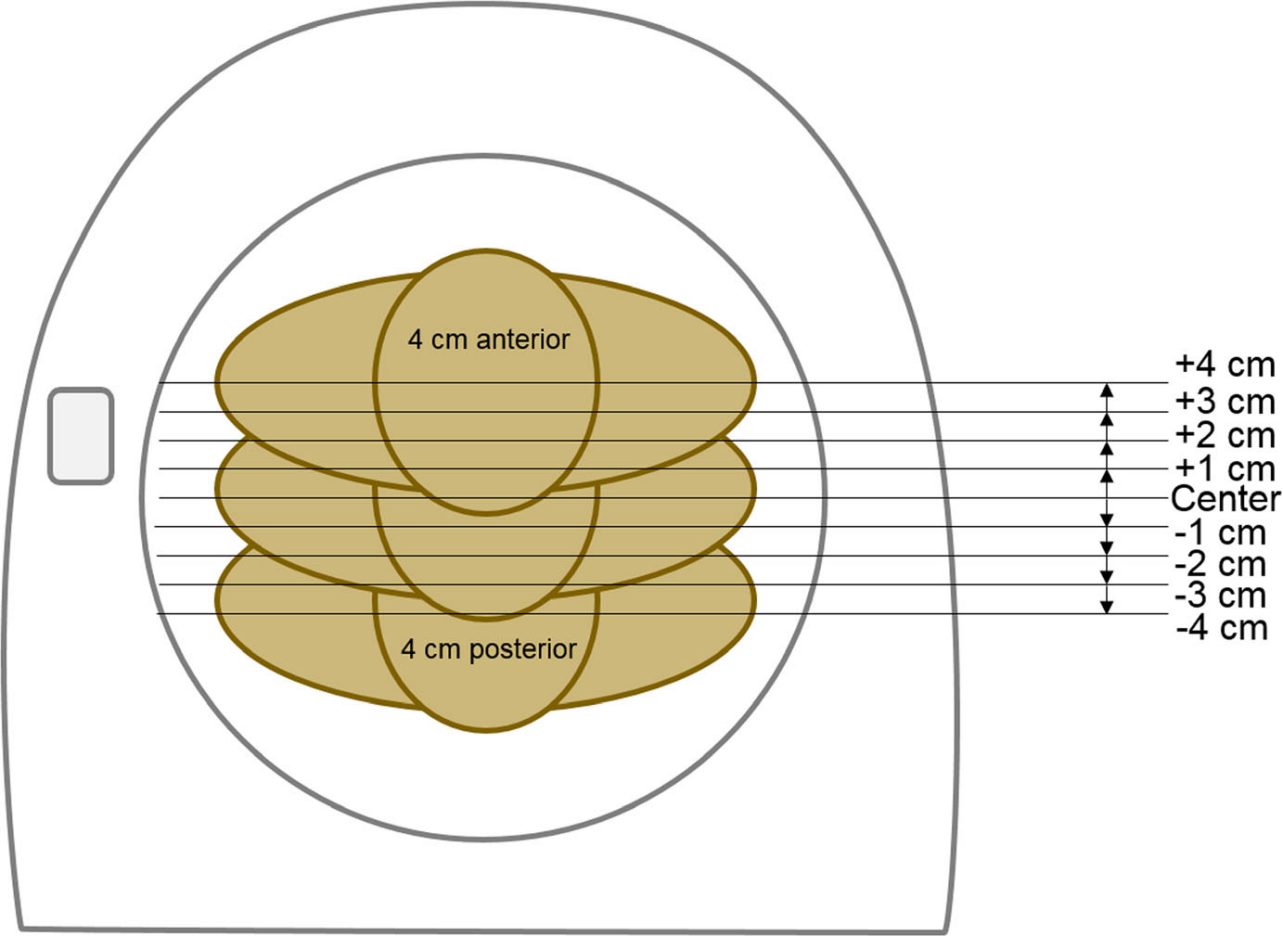
One centered scan and eight shifted scans were acquired for each postmortem subject. The table was shifted away from center in increments of 1‐cm in the anterior and posterior directions, producing maximum shifts of 4 cm in the anterior and posterior directions.

Three embalmed adult postmortem subjects were utilized in this work, with weight categories ranging from normal to obese (body mass index 23.5–34.2 kg/m^2^). All subjects were scanned on a 320‐slice CT scanner (Aquilion ONE, Canon Medical Systems, Otawara, Japan) using a clinical CAP CT protocol acquired at 120 kVp, helical mode, 0.89 pitch, 0.5 mm × 64 detector configuration, and automatic TCM with a noise target level of 12.5 SD. A centered scan was acquired with the lasers aligned at the midline of the subject's torso in both the lateral and vertical planes. Eight shifted scans were acquired with the table shifted 1, 2, 3, and 4 cm in both the anterior and posterior directions. The table shifts were selected based on typical clinical mis‐centering distances identified in the retrospective investigation of this work. The table was shifted away from isocenter using the table‐mounted table‐controls. One centered scan and eight shifted scans were performed on each postmortem subject, as demonstrated in Fig. 4, acquired once without and once with the PCS enabled.

For each scan, the scanner‐reported volumetric CT Dose Index (CTDI_vol_) was recorded. Electronic calipers on the viewing software were utilized to measure the widest lateral dimension in the pelvis on the localizer radiograph image in order to demonstrate magnification as a function of table shift. The tube current for each slice was extracted from the DICOM header and plotted along the z‐axis as a function of the table shift.

Optically stimulated luminescent dosimeters (OSLDs) were inserted into the lungs, breasts, liver, stomach, colon, uterus, and ovaries and placed on the skin regions included in the scan range utilizing the methodology described by Griglock et al.^17^ Organ doses were measured at each table position and corrected for energy and scatter response.^17^

### 2.3 Statistical analysis

A student's *t*‐test was used to assess differences in mis‐centering distances between the male and female patients in the study. Pearson correlation coefficients (*R* ^2^) were used to evaluate the relationship between patient effective diameter and mis‐centering distances in the vertical and lateral directions. A student's *t*‐test was also used to test whether CTDI_vol_ and mA values at each vertical table position were significantly different from values recorded at the center of the gantry. Percent differences were calculated at each table height position relative to the organ dose measurements acquired at the central position. Pearson correlation coefficients (*R* ^2^) were used to evaluate the relationship between table position and organ doses. The confidence levels of 95% were calculated, and a two‐tailed *P *< 0.05 was considered to indicate statistically significant differences.

## 3 RESULTS

### 3.1 Clinical frequency and magnitude of positioning errors

**Figure 5:**
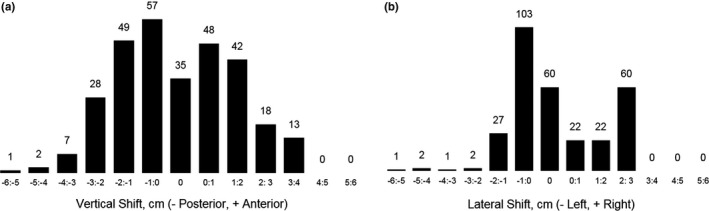
Histogram with number of patients shifted from the center of the CT gantry in the (a) vertical and (b) lateral directions in categorical bins of 1‐cm. Shifts labeled 0‐cm include patients shifted <0.5 mm away from the center of the gantry.

Correct patient centering (<0.5 mm shift from the center of the gantry) occurred for 12% of patients in the vertical direction (*n* = 35) and for 20% of patients in the lateral direction (*n* = 60). Vertical mis‐centering was >0.5 mm in 88% of patients, >0.5 cm in 73% of patients, and >1 cm in 53% of patients. The maximum vertical shift occurred at a posterior shift of 5.4 cm. Lateral mis‐centering was >0.5 mm in 80% of patients, >0.5 cm in 54% of patients, and >1 cm in 21% of patients. The maximum lateral shift occurred at 5.2 cm to the patient's left side. Figure 5 displays histograms of the frequency and magnitude of mis‐centering from the center of the gantry in both the vertical and lateral directions in increments of 1‐cm.

There was no significant difference in vertical (Mean: −0.07 cm males, −0.12 cm females, *P* = 0.83) or lateral (Mean: −0.02 cm males, 0.01 cm females, *P* = 0.84) mis‐centering distances between male and female patients. Also, no correlations were observed between patient effective diameter and mis‐centering magnitude in the vertical (*R* ^2^ = 0.03) or lateral (*R* ^2^ = 0.002) directions.

### 3.2 Magnification and dose output

**Figure 6:**
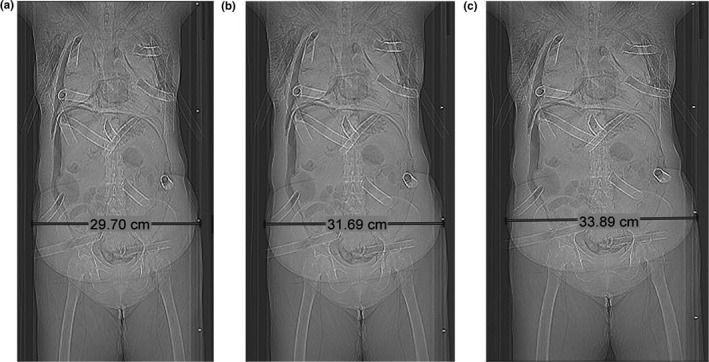
Localizer radiograph images of Subject 3 (a) shifted 4 cm posteriorly, (b) centered, and (c) shifted 4 cm anteriorly.

**Figure 7:**
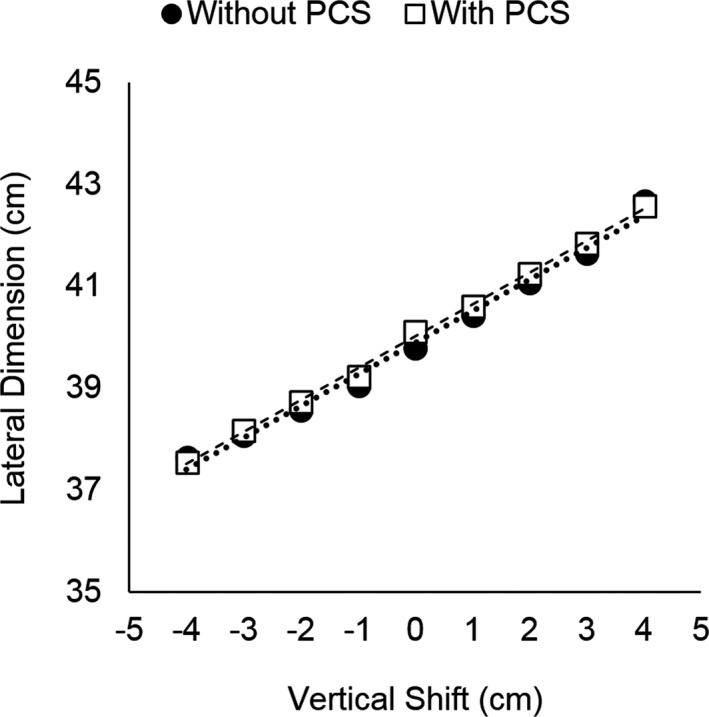
Lateral dimension of Subject 3 at the widest diameter in the pelvis region acquired with and without the positioning compensation system at each vertical shift.

The magnification effect from the projection nature of the localizer acquisition resulted in an incorrect estimate of the actual patient size. Figures 6(a), 6(b), and 6(c) display localizer radiograph images and lateral caliper measurements of Subject 3 scanned at a 4‐cm posterior shift, centered, and at a 4‐cm anterior shift, respectively. When shifted 4‐cm posteriorly, the lateral measurements reduced from 31.69 cm to 29.70 cm (6.3% decrease), and when shifted 4‐cm anteriorly, the measurements increased from 31.69 to 33.89 (6.9% increase). Figure 7 shows maximum pelvis lateral dimensions of Subject 3 measured from each localizer radiograph image acquired at all nine table positions, showing a significant linear increasing trend with table height (*R* ^2^ = 0.99, *P* < 0.005). Similar trends were observed for Subjects 1 and 2, with the lateral dimension decreasing by 6.8% and 5.4% when shifted 4‐cm posteriorly for Subject 1 and Subject 2, respectively, and increasing by 6.1% and 7.3% when shifted 4‐cm anteriorly for Subject 1 and Subject 2, respectively. All three subjects demonstrated the same linear increase in patient size as the subjects were positioned closer to the x‐ray tube (*R* ^2^ > 0.98).

**Figure 8:**
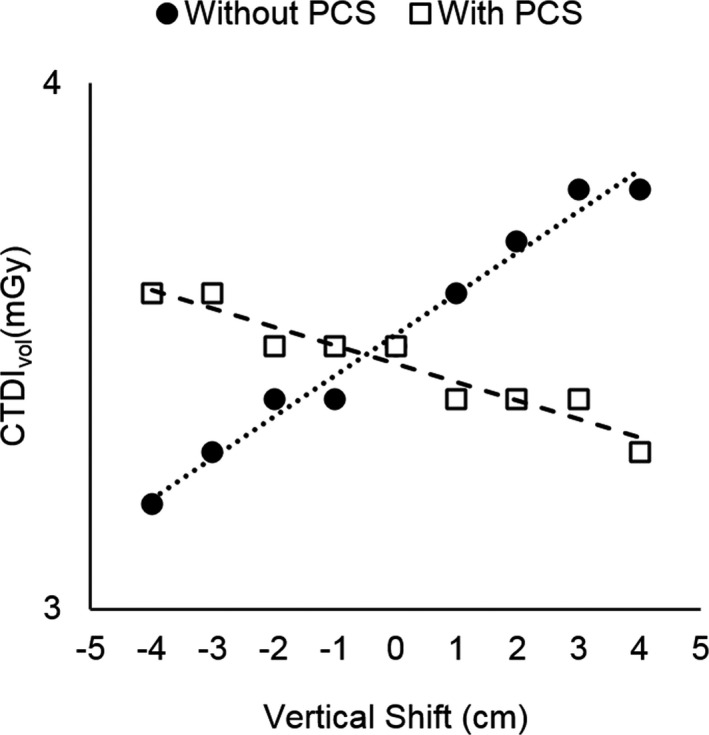
Volumetric computed tomography dose Index (CTDI_vol_) of Subject 3 scanned with and without the positioning compensation system at each vertical shift.

CTDI_vol_ values also increased linearly with anterior table shift of Subject 3 (*R* ^2^ = 0.98, *P* < 0.005), shown in Fig. 8. When the table was shifted 4 cm posteriorly from center, the CTDI_vol_ reduced from 3.5 to 3.2 mGy (8.5% decrease), and when the table was shifted 4 cm anteriorly from center, the CTDI_vol_ increased from 3.5 to 3.8 mGy (8.5% increase). Similar trends were also observed for Subjects 1 and 2 (*R* ^2^ > 0.98).

When the PCS was utilized, similar trends in magnification were observed, with the lateral dimension of all subjects increasing linearly with anterior table shift (*R* ^2^ > 0.96, *P *< 0.005), as shown in Fig. 7. However, CTDI_vol_ values experienced a different behavior, where CTDI_vol_ instead decreased with increasing table height for all subjects (*R* ^2^ > 0.92, *P* < 0.005), shown in Fig. 8 for Subject 3. When the table was shifted 4‐cm posteriorly, the CTDI_vol_ increased from 3.5 to 3.6 mGy (2.8% increase), and when the table was shifted 4‐cm anteriorly, the CTDI_vol_ decreased from 3.5 to 3.3 mGy (5.7% decrease).

**Figure 9:**
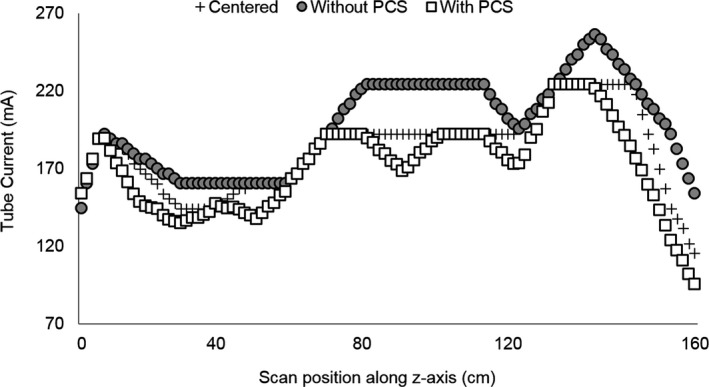
Tube current values along the z‐axis of Subject 3 for a centered scan and scans shifted 4 cm anteriorly acquired with and without the positioning compensation system.

Significant differences were observed in tube current values delivered at different vertical shifts compared to the reference position (*P* < 0.005). Figure 9 displays the TCM map along the z‐axis of Subject 3 for the centered scan and the scan shifted 4 cm anteriorly both with and without the PCS. The PCS brought the tube current values closer to those used when the subject was centered, reducing the absolute percentage difference between the shifted and centered scans (mean 6%, maximum 19%) compared to when the PCS was not used (mean 9%, maximum 34%).

### 3.3 Organ dosimetry

**Figure 10:**
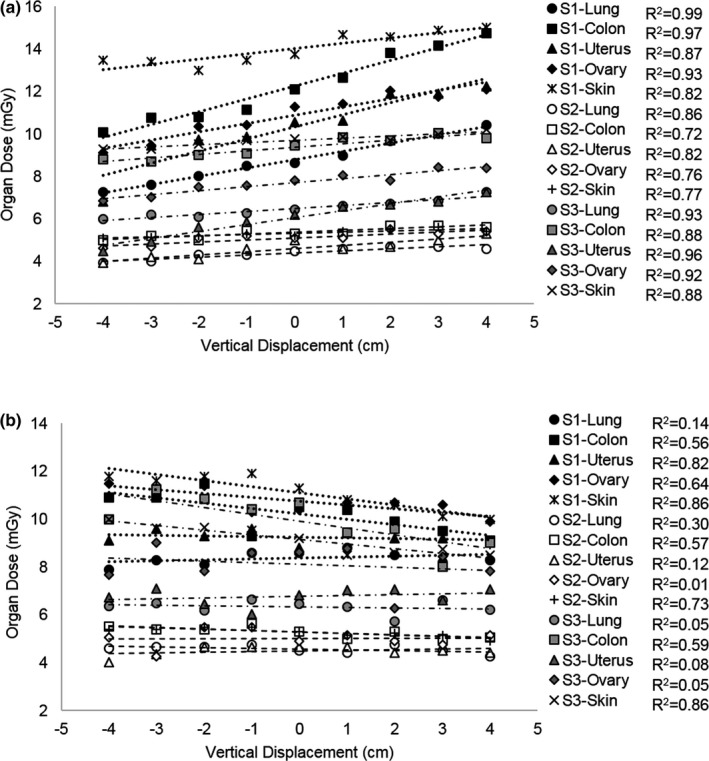
Organ doses for the lung, colon, uterus, ovary, and skin as a function of vertical shift from the center of the gantry acquired (a) without the positioning compensation system (PCS) and (b) with the PCS in Subject 1 (S1), Subject 2 (S2), and Subject 3 (S3).

**Figure 11:**
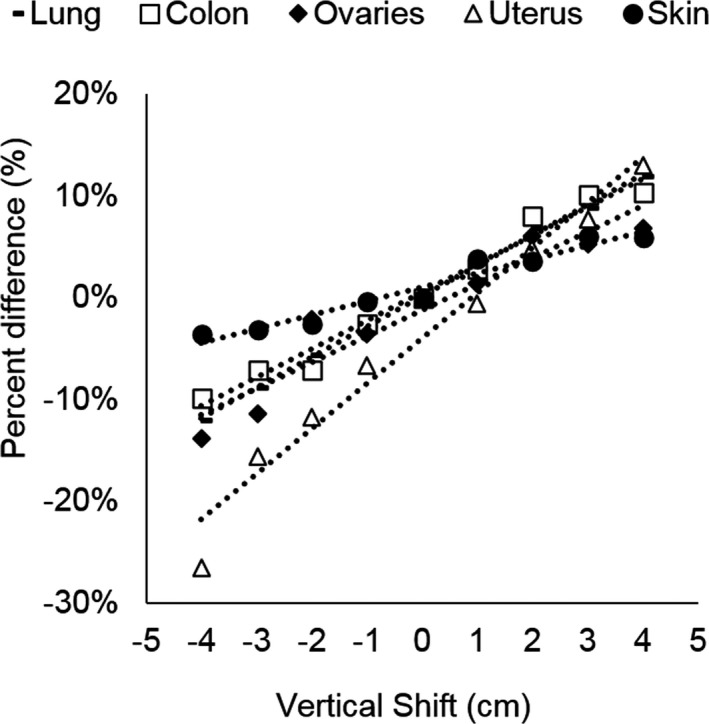
Organ dose percent differences averaged over all three subjects as a function of vertical shift from the center of the gantry for the lung, colon, ovaries, uterus, and skin acquired without the positioning compensation system.

**Figure 12:**
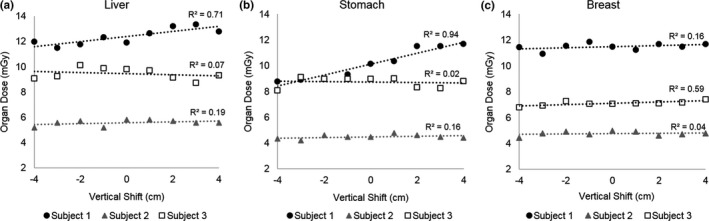
Organ doses for the (a) Liver, (b) Stomach, and (c) Breast for Subjects 1, 2, and 3. The liver and stomach showed strong linear correlations for Subject 1, but not for Subjects 2 and 3. The breast showed a strong linear correlation for Subject 3, but not for Subjects 1 and 2.

All organ dose measurements are presented in milligray (mGy) in Table 1. When the PCS was not used, significant correlations were found between organ doses and vertical table shifts for all subjects in five out of eight organs including the lung, colon, uterus, ovary, and skin, demonstrating an increase in organ dose with increasing vertical shift (*R* ^2^ = 0.73–0.99, *P* < 0.005), shown in Fig. 10(a). For these five organs, organ dose differences relative to the centered position ranged from −35.0% to 22.0%. Figure 11 displays organ dose percent differences in these five organs, averaged across the three postmortem subjects (Standard Deviation 0.008–0.099) measured at each table shift position ranging from 4 cm posterior to 4 cm anterior (*R* ^2^ = 0.90–0.99, *P* < 0.005). The liver, stomach, and breast organ doses had smaller relative organ dose differences, ranging from −13.0% to 15.0%, compared to the other five organs mentioned above. Strong linear correlations were observed for the liver (*R* ^2^ = 0.71) and stomach (*R* ^2^ = 0.94) in Subject 1 and for the breast (*R* ^2^ = 0.59) in Subject 3, but correlations were weak for these organs in the other subjects (*R* ^2^ = 0.01–0.50), as shown in Fig. 12.

**Table 1:** Organ doses (mGy) measured in Subjects 1, 2, and 3 scanned with and without PCS

	Without positioning compensation system									With positioning compensation system								
	−4	−3	−2	−1	0	1	2	3	4	−4	−3	−2	−1	0	1	2	3	4
Subject 1																		
Lung	7.3	7.6	8.0	8.5	8.6	9.0	9.7	10.0	10.4	7.9	8.3	8.1	8.6	8.6	8.8	8.5	8.2	8.3
Breast	11.4	11.0	11.6	11.8	11.5	11.2	11.7	11.5	11.7	12.1	12.0	11.8	11.8	11.0	10.8	10.3	10.2	9.5
Liver	12.0	11.5	11.8	12.3	11.9	12.7	13.2	13.3	12.8	8.6	8.6	9.5	9.7	9.2	8.8	9.0	9.2	9.2
Stomach	8.8	8.9	9.0	9.3	10.1	10.3	11.5	11.5	11.7	9.8	9.6	9.5	9.8	9.6	9.4	9.0	9.2	8.4
Colon	10.1	10.7	10.8	11.2	12.1	12.7	13.8	14.1	14.8	10.9	10.9	11.5	9.3	10.5	10.4	9.9	9.5	9.1
Uterus	6.8	9.5	9.7	9.9	10.5	10.6	11.9	11.9	12.2	9.1	9.6	9.3	9.6	8.8	9.4	9.2	9.2	9.1
Ovary	9.1	9.5	10.3	10.4	11.3	11.4	12.0	11.8	12.1	11.5	11.3	11.4	10.3	10.4	10.7	10.7	10.6	9.9
Skin	13.5	13.4	13.0	13.5	13.7	14.7	14.6	14.9	15.0	11.8	11.6	11.8	11.9	11.3	10.8	10.6	10.1	10.0
Subject 2																		
Lung	3.9	4.0	4.3	4.3	4.5	4.6	4.7	4.7	4.6	4.6	4.7	4.7	4.7	4.5	4.4	4.7	4.5	4.3
Breast	4.4	4.8	4.9	4.7	5.0	4.9	4.6	4.7	4.8	5.3	5.3	5.2	4.7	4.8	4.3	4.6	4.2	4.0
Liver	5.2	5.6	5.7	5.2	5.8	5.8	5.7	5.6	5.6	5.6	6.2	5.9	5.8	5.3	5.4	5.5	4.6	4.8
Stomach	4.3	4.2	4.6	4.5	4.5	4.8	4.6	4.5	4.4	5.4	5.1	4.8	4.8	4.4	4.3	4.2	4.1	3.6
Colon	5.0	5.2	5.0	5.5	5.3	5.3	5.7	5.7	5.6	5.5	5.4	5.4	5.6	5.3	5.0	5.3	5.0	5.1
Uterus	3.9	4.2	4.1	4.6	5.0	4.6	4.7	5.0	5.3	4.0	4.3	4.7	4.7	4.7	4.7	4.4	4.5	4.4
Ovary	4.6	4.7	5.1	5.2	5.1	5.1	5.5	5.3	5.4	5.1	4.3	5.5	5.5	4.9	5.1	4.9	4.7	5.1
Skin	5.1	5.1	5.2	5.3	5.2	5.4	5.5	5.6	5.4	5.4	5.4	5.5	5.5	5.3	5.1	5.2	5.1	4.9
Subject 3																		
Lung	6.0	6.2	6.1	6.3	6.4	6.6	6.7	6.9	7.3	6.4	6.5	6.2	6.7	6.5	6.3	5.7	6.6	6.2
Breast	6.8	6.9	7.3	7.1	7.1	7.1	7.1	7.2	7.4	7.6	7.4	7.3	6.8	7.1	6.9	6.2	6.5	6.5
Liver	9.1	9.3	10.1	9.9	9.8	9.7	9.1	8.7	9.3	9.4	9.3	9.3	8.9	8.5	8.5	7.7	8.2	7.4
Stomach	8.1	9.1	9.0	9.0	9.0	9.0	8.3	8.3	8.8	8.0	8.8	8.5	8.5	8.1	6.3	7.7	7.8	7.8
Colon	8.8	8.7	9.0	9.1	9.5	9.8	9.7	10.0	9.8	10.0	11.2	10.8	10.4	10.7	9.4	9.6	8.0	9.0
Uterus	4.5	4.9	5.6	5.9	6.2	6.6	6.7	6.8	7.3	6.7	7.1	6.5	6.0	6.8	7.0	7.1	6.6	7.1
Ovary	6.8	7.0	7.5	7.6	7.8	8.1	7.8	8.4	8.4	7.7	9.0	7.8	8.6	8.6	8.8	6.3	8.5	7.8
Skin	9.3	9.3	9.5	9.7	9.8	9.9	9.7	10.0	10.2	10.0	9.6	9.7	9.5	9.2	8.5	8.6	8.7	8.5

**Figure 13:**
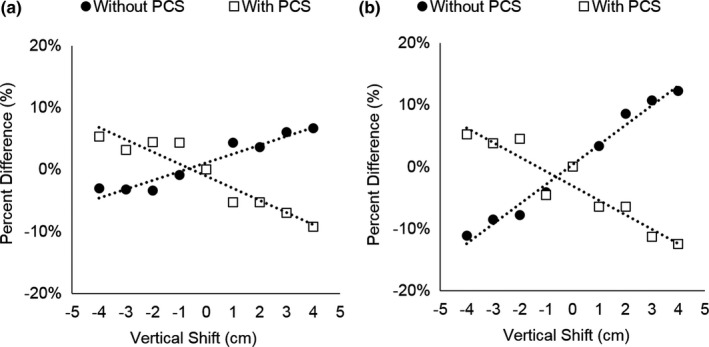
Organ dose differences averaged over all three subjects as a function of vertical shift from the center of the gantry for **(**a) the skin acquired with and without the positioning compensation system (PCS) and (b) the colon acquired with and without the PCS.

When the PCS was utilized, organ doses were observed to decrease as a function of vertical shift, as the PCS attempted to compensate for the table shift, as shown in Fig. 10(b). Significant correlations were observed between vertical shift and organ dose differences relative to organ doses measured at the center of the gantry in all three subjects for the skin (*R* ^2^ = 0.73–0.86, *P* < 0.005) and colon (*R* ^2^ = 0.90–0.98, *P *< 0.005) but did not have a strong correlation for the lungs, uterus, or ovaries (*R* ^2^ = 0.01–0.30, *P* < 0.005). Figures 13(a) and 13(b) display organ dose differences averaged over all three subjects with and without the PCS for the skin and colon, respectively.

## 4 DISCUSSION

The frequency of clinical mis‐centering of patients undergoing CT examinations in our institution was slightly less than those found in other studies. For example, one study reported that 81% of patients were vertically mis‐centered by >0.5 cm,^10^ or 8% greater than our findings, while another study reported that 74% of patients were mis‐centered by >1 cm,^6^ or 21% greater than our findings. The frequency of clinical mis‐centering is likely dependent on the technologist training methods and years of experience, so it is not expected that one hospital would have the same frequency of clinical patient mis‐centering as another. In addition, it was concerning to find that 23 patients (7.7%) were mis‐centered vertically by >3 cm, as one may expect this to be detected by the technologists. However, similar findings were also reported in other studies, where one group found that 17% of their patients were mis‐centered by >3 cm,^10^ and another group reported that 22% of their patients were mis‐centered by >3 cm with maximum errors ranging from 6.6 cm posterior to 3.4 cm anteriorly.^6^ Our findings demonstrated that mis‐centering was more pronounced in the vertical direction than in the lateral direction, and showed greater prevalence for posterior mis‐centering (54% of patients) rather than anterior mis‐centering (46% of patients), in agreement with other studies.^10^

The CTDI_vol_ increased linearly with anterior table shift without PCS because magnification caused the subject to appear larger at table positions closer to the x‐ray tube, as shown in Fig. 7(a), and therefore the scanner responded by increasing tube current output. A similar trend in increasing CTDI_vol_ with increasing anterior shift was reported by the manufacturer when they scanned a phantom at different table heights.^15^ They observed the CTDI_vol_ decreased by about 7% at a 4‐cm posterior shift and increased by about 10% at 4‐cm anterior shift. This is similar to our findings of about 8.5% decrease and 8.5% increase at 4‐cm posterior and 4‐cm anterior shift, respectively. When PCS was used, the system attempted to compensate for the table shift, resulting in a decreasing CTDI_vol_ with increasing table height. The manufacturer also demonstrated that their CTDI_vol_ values increased by about 5% at a 4‐cm posterior shift and decreased by about 3% at a 4‐cm anterior shift. This is similar to our findings of 2.8% increase and 5.7% decrease at 4‐cm posterior and 4‐cm anterior shift, respectively. The small differences in CTDI_vol_ increase and decrease between the manufacturer and our study can be due to differences in the subject being scanned. For example, the body phantom scanned by the manufacturer appeared to be much thinner than the postmortem subjects scanned in this work, and the scan techniques were likely different. In general, the linear trends observed for CTDI_vol_ versus table shift with and without PCS were in agreement with those reported by the manufacturer.

It can be argued that we would expect for the PCS system to correct the CTDI_vol_ at all table shifts to match the CTDI_vol_ produced at center. However, neither we nor the manufacturer observed this. In fact, it appears that rather than normalizing the values to be consistent, the system slightly overcompensated the output in the opposite direction, resulting in a decreasing trend of CTDI_vol_ with table height. However, both this study and the manufacturer found that the change in CTDI_vol_ was greater without PCS (range 7%–10%) than with PCS (range 2.8%–5.7%).

**Figure 14:**
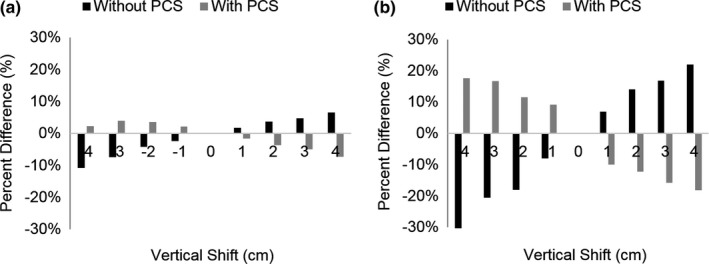
(a) Average and (b) maximum organ dose differences for all organs and postmortem subjects at each vertical shift for scans acquired with and without the positioning compensation system.

Although the PCS did not normalize CTDI_vol_ and organ doses to be equivalent to the scan acquired at the center of the gantry, it was able to provide smaller organ dose differences. For example, the PCS reduced the average dose difference at 4 cm posterior shift from 11% to 2%, shown in Fig. 14(a), and also reduced the maximum dose difference among all subjects and organs from 35% to 18%, shown in Fig. 14(b).

In addition to the magnification in the localizer radiograph, there are other complex mechanisms in the scanner that affect patient dose, such as the bowtie filter. When the center of mass is shifted away from the gantry, some organs will be shifted towards the center of the gantry, and therefore will experience an increase in dose due to the bowtie filter allowing maximum beam intensity at the central region of the beam, as shown in Fig. 1.

While the PCS alters the tube current to correct for patient magnification, it does not modify the shape or position of the bowtie filter to account for mis‐centered patients. It is possible that these competing mechanisms of TCM, bow‐tie filter, and PCS affect the output and organ doses in different ways and therefore lead to nonlinear trends and poor correlations between organ dose and vertical shift for the lung, ovaries, and uterus.

Correlations of organ dose difference as a function of table height were strong (*R* ^2^ > 0.73) for the lung, colon, uterus, ovary, and skin in all three postmortem subjects, but correlations were weak for the liver and stomach in Subjects 2 and 3 (*R* ^2^ < 0.5), and for the breast in Subjects 1 and 2 (R^2^ < 0.5). A reason for this may be due to the fact that organs that received strong correlations were located on both the left and right side of the subject anatomy, and therefore organ doses were measured in both the left and right side of the subject anatomy. However, doses were only measured on the right side of the anatomy for the liver and on the left side of the anatomy for the stomach. This may play an effect in the dose distribution due to the bowtie filter as the x‐ray tube rotates around the gantry as shown in Figs. 1(b) and 1(c). The breasts are bilateral, and doses were measured for both the left and right sides. However, the breasts are located peripherally in the patient anatomy, potentially being scanned with a different dose profile due to the bowtie filter as shown in Figs. 1(b) and 1(c).

The majority of our patients were mis‐centered posteriorly by <1 cm (*n *= 57, 19%). At this shift, we may expect relative organ dose differences up to 8%, as observed in this study. For the three patients mis‐centered >4 cm posteriorly, this study observed organ dose differences as high as 35%, with likely greater differences at greater shifts.

There are limitations to this study. The first is that this study did not measure organ doses for lateral shifts. Mis‐centering in the lateral direction will also produce magnification in the lateral localizer radiograph, affecting the TCM output. Therefore, it is expected that a lateral shift would also affect organ doses, with effects depending on whether the patient was shifted towards or away from the stationary x‐ray tube during the acquisition of the lateral localizer radiograph, as well as distribution of organs within the anatomy. We chose to focus on reporting the effects of vertical mis‐centering as these table shifts are more pronounced and prevalent, as found in the first part of this work as well as in other studies. Furthermore, shifting bilateral organs towards or away from center would introduce additional complex effects due to the bowtie filter, with one side of the organ experiencing a different effect than the other side. Second, this study did not evaluate the effect of mis‐centering on image quality. Other studies have shown that mis‐centering has an effect on both patient dose and image quality. For example, a study by Toth et al.^6^ reported that a vertical shift of 6 cm increased image noise by up to 30% and a study by Kaasalainen et al.^9^ reported that a vertical shift of 6 cm increased image noise by up to 28%. However, both of these studies conducted noise measurements in phantoms, and have not evaluated the effect of patient mis‐centering on diagnostic image quality.

## 5 CONCLUSION

In conclusion, this study has shown that patient mis‐centering occurs frequently in the clinic and impacts organ doses. It remains essential for technologists to strive for accurate patient positioning at the center of the CT gantry. In the events where positioning is not performed correctly, the commercially available position compensation system can automatically detect mis‐centering and modify the scan techniques to improve acquisition techniques for optimal scanner performance.

## CONFLICT OF INTEREST

Co‐author Manuel Arreola has been a recipient of research support from Canon Medical Systems, USA. All other authors have no disclosures.
